# Long-term evaluation of the success rate of different treatment modalities on MIH-affected teeth

**DOI:** 10.1007/s40368-025-01106-6

**Published:** 2025-09-11

**Authors:** K. Seremidi, K. Petroleka, N. Kraemer, S. Gizani

**Affiliations:** 1 Department of Paediatric Dentistry, School of Dentistry, National and Kapodistrian University of Athens, Athens, Greece; 2 Department of Paediatric Dentistry, Justus Liebig University, Giessen, Germany

**Keywords:** Molar-incisor hypomineralization, Restorative treatment, Extraction, Follow-up, Success rate

## Abstract

**Objectives:**

The objective is to report on long-term success of treatment modalities of MIH-affected teeth and correlate them with patient- and teeth-related factors.

**Methods:**

A retrospective cohort study on MIH teeth involving data collection from the patients’ dental records regarding initial treatment performed and evaluation of treatment success and need for re-treatment up to 24 months post-treatment. Fisher’s exact test and non-parametric Cuzik trend test were used to test changes in treatment and multinomial logistic analysis to investigate the effect of patient and tooth-related characteristics. Analyses were performed using STATA 18.

**Results:**

The sample consisted of 38 patients, with a mean age of 9 years at presentation. The vast majority had all four molars affected and 30% had up to two incisors. More than half of MIH teeth were severely affected (57%), showed enamel breakdown (57%) and were decayed (74%). The most common treatments performed were stainless steel crowns (34%) and composite resin restorations (27%). Treatment was successful in most cases up to 24 months post-treatment (p-values 0.05). Up to 12 months, restorations in eight teeth were rated with a Charlie/Delta mainly due to loss of marginal integrity and discoloration. Repeat of the same treatment was performed in 13 teeth, with most repeats being for sealants and preventive resin restorations. No significant correlation existed between the outcome and demographic characteristics and clinical features.

**Conclusions:**

Type of treatment is directly associated with the severity of hypomineralization, with both direct and indirect restorations showing equally high success rates. They can therefore be considered effective restorations with acceptable clinical performance.

## Introduction

Molar incisor hypomineralisation (MIH), is a qualitative developmental defect, of multifactorial origin with a genetic component, that affects permanent molars (Bussaneli et al., 2019). Affected teeth present unique characteristics such as post-eruptive structure loss, pulpal inflammation and hypersensitivity (Elhennawy and Schwendicke, 2016), characteristics that become worse in high caries risk patients (Fresen et al. 2024). Over the past decades, guidelines have been developed to give clinicians the chance to expand from scientific evidence towards better clinical practice. Guidelines highlight that restoring hypomineralised teeth is challenging, not only due to structural alterations that cause reduced mechanical resistance of the affected tissue (Pitiphat et al., 2014), but also due to the increased treatment requirements caused by the extensive restorations, the great number of visits, and the regular monitoring that is necessary.

Different approaches have been introduced in the literature depending on the extent and the severity of the lesions present, stage of dental development, presence of hypersensitivity and patient-related factors, such as caries risk, oral hygiene level, cooperation and their preferences and expectations (da Cunha Coelho et al. 2019). Fissure sealants are commonly used in mild lesions where sensitivity is not prominent and there is usually no enamel breakdown (Lygidakis et al. 2022), whilst restorative treatment of moderately and severely affected teeth range from simple composite resin restorations to stainless steel crowns (SSCs) aiming at sealing affected tissue to prevent further hard tissue loss (Elhennawy and Schwendicke 2016). Extraction might be the only clinical option in severe cases; however, these cases may in turn need orthodontic treatment (Lygidakis et al. 2010; Elhennawy et al., 2016).

Lately, less invasive techniques have been incorporated for the treatment of MIH-affected teeth, especially in cases of severely affected molars or for younger patients and patients with difficulties in cooperation. The application of silver diamine fluoride has been recommended for reducing caries incidence and arresting progression in cavitated lesions (Crystal and Niederman, 2019; Al-Nerabieah et al. 2024; Zheng et al. 2023). It is also effective against dentine hypersensitivity, as silver ions are capable of occluding dentinal tubules through precipitation of proteins (Erbas Univerdi et al., 2024). Therefore, in some cases, it is used as an intermediate treatment option for initial pain control and hard tissue protection until the ideal time and conditions for a more definite restoration are present (Malfa et al., 2024). Within the contexts of minimally invasive dentistry, the use of SSC in a non-invasive way, where only unsupported hypomineralised structures and previous unsatisfactory restorations are removed without any further tooth preparation, has been also supported (de Farias et al. 2022). These procedures aim at protecting hypomineralised tissue and reducing sensitivity whilst ensuring children’s cooperation and meeting patient’s preferences in an attempt to improve oral health-related quality of life.

The ideal restorative material is yet to be identified as long-term outcomes of different treatment protocols on MIH-affected teeth have not yet been documented extensively (Elhennawy and Schwendicke 2016). To date, the evidence is inconclusive, as there is insufficient high-quality scientific evidence to establish a definitive clinical protocol. Considering the 11-fold greater amount of treatment and three times more re-treatment needs reported for MIH-affected teeth (Kotsanos et al. 2005), the need for more focussed studies comparing various treatment protocols is clearly emphasised to develop standardised management protocols to help clinicians make treatment decisions. Therefore, the aim of the present study was to report on the long-term success of different treatment modalities of MIH-affected teeth in children seeking dental care in a university clinical setting. Further objectives were to:

- report on the prevalence of various clinical features of MIH-affected teeth
- evaluate existing restorations and report re-treatment needs
- correlate treatment protocols with patient and teeth-related characteristics.

## Materials and methods

### Study design and setting

A retrospective cohort study was undertaken, on patients diagnosed with varying severity of MIH-affected teeth, according to the updated EAPD criteria (Lygidakis et al. 2022). It involved data collection from patients’ dental records regarding the initial treatment performed on MIH-affected teeth, by way of the clinical photographs and radiographs.

The research protocol was submitted and approved by the Ethics Committee of the School of Dentistry, National and Kapodistrian University of Athens, Greece (No 572/16.02.2023).

### Participants

The sample was derived from the patients seeking dental care in the clinics of the Department of Paediatric dentistry (NKUA) between January 2016 and December 2023. It consisted of all patients with at least one MIH-affected first permanent molar  that met the following inclusion criteria:

- Patients with full medical and dental records
- Patients with a complete record of clinical photographs and radiographs, pre- and post-operatively and follow-up sessions
- Patients attending the clinic on a 6-month basis
- Patients with a minimum follow-up of 12 months post-operatively.

Patients with incomplete dental records, no pre-operative photographs or photographs of poor quality and those with missing clinical photographs and radiographs in any of the follow-ups were excluded from the study.

All treatments had been performed under local anaesthesia (articaine) in the paediatric dental clinic by five different operators, following a strict protocol based on the EAPD guidelines (Lygidakis et al. 2022). The operators, that were post-graduate students, were constantly supervised by an Assistant Professor at the Department and they were assessed and calibrated on a monthly basis. Treatments, that ranged from non-invasive (e.g. sealants) to invasive techniques (e.g. SSC and extractions), were decided based on lesion (e.g. severity, presence of caries and post-eruption enamel breakdown) and patient-specific characteristics (dental status, age and cooperation).

### Data collection and measurements

Data regarding demographic characteristics (gender, age, medical history), oral hygiene and dietary habits were collected from patients’ records. From dental records, data regarding oral hygiene and caries experience at first presentation (baseline findings) were also collected, followed by evaluation of MIH-affected teeth. Number of posterior and anterior teeth and surfaces affected, and type and severity of lesions were noted according to the definitions provided on the updated EAPD guidelines (Lygidakis et al. 2022). Finally, radiographic evaluation of pulp involvement and root developmental status of each MIH-affected tooth was also undertaken.

Following baseline findings, initial treatment performed on each tooth was recorded in detail along with its clinical acceptance at each subsequent follow-ups at 6, 12, 18 and 24 months. Each restoration was evaluated for its anatomical form, marginal adaptation, marginal discoloration, retention and secondary caries formation using the modified USHPH (Bayne and Schmalz 2005) criteria. Restorations were considered successful if all parameters were rated as sufficient and clinically acceptable (alpha/bravo) and as a failure if at least one parameter was rated as insufficient (Charlie/delta).

All evaluations were performed by two independent, qualified paediatric dentists, not involved in the initial treatment of the patient (neither as an operator nor as a supervisor). Evaluators were previously calibrated on clinical photographs taken from MIH-affected teeth selected by another staff member, until an inter-examiner reliability of k > 0.9 was reached.

### Statistical analysis

Sample selection was made at the patient level, whilst analysis was performed at tooth level, evaluating success of treatment undertaken for each affected tooth. Distributions of demographic characteristics and various clinical features were described by frequencies and percentages for categorical variables and mean ± standard deviation (SD) for continuous variables. Prevalence of the clinical features of MIH-affected teeth at baseline was expressed as percentages. Success rates, restorations evaluation and changes in treatment throughout the follow-up periods were investigated using Fisher’s exact test and non-parametric Cuzick trend test. Multinomial logistic analysis was performed to investigate the relationship between treatment outcome and specific patient and tooth-related characteristics such as gender, age, number of molars affected, co-existence of lesions in incisors, type and severity of lesion and pulp involvement. Two-tailed *p* values were reported, with a *p* value < 0.05 considered as statistically significant. All analyses were performed using STATA 18 (StataCorp LP, College Station, Texas).

## Results

### Participants’ characteristics

The sample consisted of 38 patients, 20 females and 18 males, with a mean age of 9 years (± 1.8) at first visit. More than 2/3 were referred by a general practitioner (69%) with pain upon drinking hot (16%) and cold (40%) beverages and upon chewing (5%). Primary chief complaints reported by the parents were the presence of broken (13%), carious (29%) and discoloured teeth (16%).

Participants in their majority were healthy (97%), of Greek nationality (74%) and born after full-term pregnancy (89%). They reported brushing alone (97%), at least once daily (77%), with a manual toothbrush (100%) and a fluoridated toothpaste (96%). More than half (55%) reported consuming sugary snacks almost every day.

### Descriptive data

At baseline, 87% of the participants were in mixed dentition, with 52% having moderate oral hygiene and 32% poor. Overall DMFT/dmft value was 3 ± 2.3, with 60% presenting with at least one carious tooth, all teeth were included (ICDAS > 4).

**Fig. 1:**
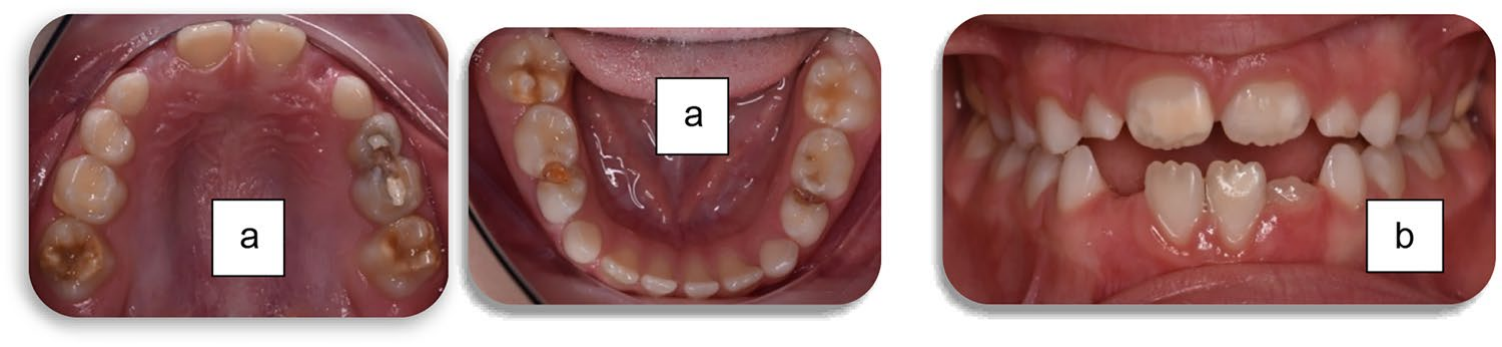
Clinical photographs of MIH patients from the sample showing a. lesions of various severity in permanent molars and b. lesions in anterior teeth

**Fig. 2:**
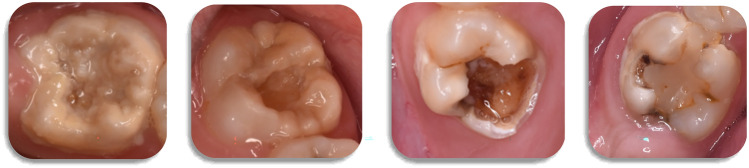
Clinical photographs of severely affected first permanent molars, presenting combinaction of lesions including brown opacities, post-eruptive enamel breakdown and atypical restorations

**Fig. 3:**
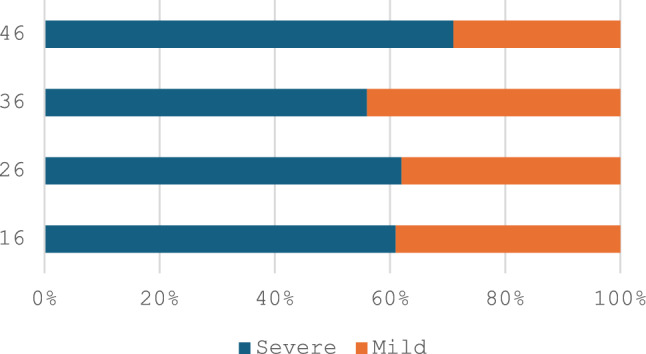
Distribution of lesion severity in MIH-affected molars

Regarding MIH-affected teeth, at patient level, 79% had all four first permanent molars affected (*n* = 30) (Fig. 1), 10% had three molars affected and only 10% of the patients had only one or two affected molars. Forty seven percent (*n* = 18) of the participants did not have any incisors affected, 30% (*n* = 12) had up to two with the remaining 21% having > 3 incisors involved (Fig. 1). At tooth level, 194 teeth were affected, 141 molars and 53 incisors. From the affected molars, 73 were from the upper arch and the remaining 68 from the lower with the distribution between quadrants in each arch being equal. Overall, more than half of MIH-involved molar teeth were severely affected (Fig. 2), with the distribution of prevalence between upper and lower arches not differing significantly (Fig. 3). Half of the patients also reported hypersensitivity (*n* = 19).

**Fig. 4:**
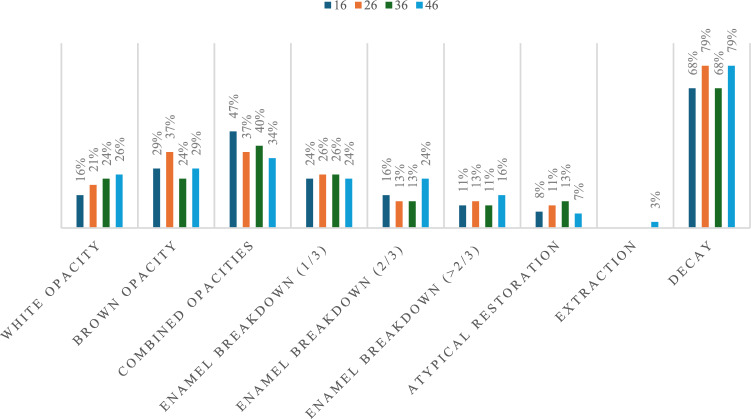
Distribution of different characteristics of MIH-affected teeth

When considering the distribution of specific characteristics of affected molars (*n* = 141), 92% (*n* = 126) had all four surfaces affected, with more than 85% (*n* = 120) presenting opacities, more than 50% (*n* = 73) enamel breakdown and around 10% (*n* = 12) atypical restorations. Seventy four percent (*n* = 102) were also decayed (Fig. 4). Both white-opaque and yellow–brown opacities were detected in more than 1/3 of affected teeth, whilst breakdown up to 1/3 of the surface was noticed in most MIH-affected molar teeth. Higher frequencies of the combination of opacities were found for the upper molars, whilst more severe breakdown, atypical restorations and extractions were noted in lower molars, with the differences not showing statistical significance. All incisors presented mild white-opaque lesions in the buccal surfaces.

### Outcome data

**Fig. 5:**
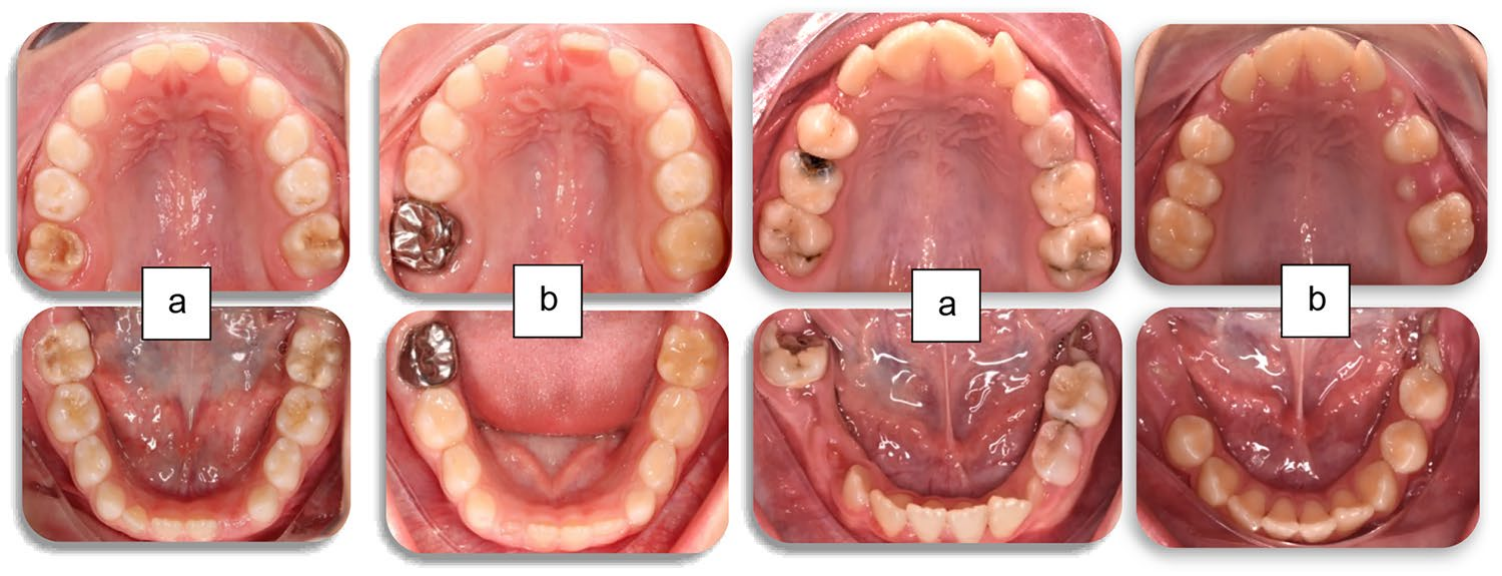
Clinical photographs of full mouth rehabilitation of two severe MIH-cases: a. at initial presentation and b. after completion of treatment

**Fig. 6:**
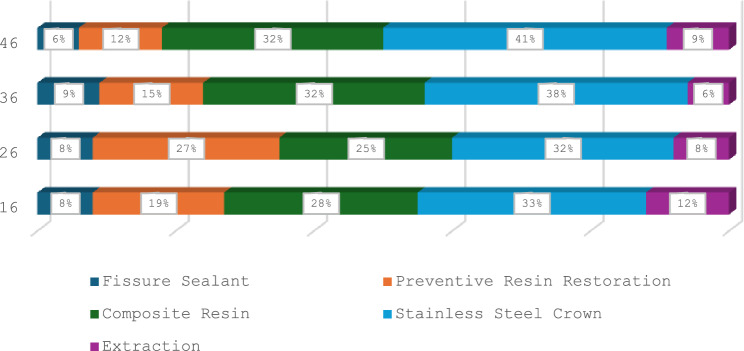
Distribution of initial treatment between MIH-affected teeth

From all the affected molars, 89% had undergone treatment, whilst the remaining 15 teeth were only monitored as they were partially erupted. From the MIH-affected molars, 26% underwent micro-invasive treatment, and 74% underwent restorative treatment (Fig. 5). Regarding micro-invasive treatment, 30% had fissure sealants, whilst 70% had preventive resin restorations due to micro-cavitated lesions extending only into enamel. The most common type of invasive/restorative treatment performed (Fig. 6) was SSC (34%), followed by composite resin restorations (27%). Extractions were performed in only 8% of the cases. From the more severely affected molars, 17% were treated with vital pulp therapy and 4% had undergone root canal treatment. Distribution of treatment amongst MIH-affected molars did not reveal any significant differences.

No interventions were undertaken for the MIH-affected anterior teeth, as they were of mild severity and did not pose any aesthetic problems.

### Main results

Table 1 presents the evaluation of direct restorations performed in the MIH-affected molars at the four follow-up periods up to 24 months post-treatment. No significant differences were observed (*p* values > 0.05), with most treatments evaluated as successful. Specifically, up to 12 months, restorations in eight teeth (21%) were rated as unsatisfactory (Charlie/Delta) mainly due to loss of marginal integrity and discoloration. During all follow-ups, only one MIH-affected tooth failed due to secondary caries formation at 12 months. The high success rate of the coronal seal reflects the 100% success rates of all vital and non-vital pulp therapies.

**Table 1:** Evaluation of treatment undertaken for each MIH-affected tooth at follow-ups

	Follow-up (months: mean; SD) 17.5 (6.25)					
	6 (*n* = 126)	12 (*n* = 126)	18 (*n* = 66)	24 (*n* = 59)	*p value*^1^	Trend *p* value
Overall outcome						
**Success**	**97 (122)**	**97 (122)**	**100 (66)**	**100 (59)**	**0.96**	**0.71**
**Failure**	**3 (4)**	**3 (4)**	**0 (0)**	**0 (0)**		
**16 (*n*, %)**						
Alpha/Bravo	29 (94)	28 (93)	15 (100)	13 (100)	0.95	0.63
Charlie/Delta	2 (6)	2 (7)	0 (0)	0 (0)		
**26 (*n*, %)**						
Alpha/Bravo	32 (97)	32 (100)	17 (100)	15 (100)	0.99	0.88
Charlie/Delta	1 (3)	0 (0)	0 (0)	0 (0)		
**36 (*n*, %)**						
Alpha/Bravo	31 (100)	30 (97)	17 (100)	16 (100)	0.98	0.51
Charlie/Delta	0 (0)	1 (3)	0 (0)	0 (0)		
**46 (*n*, %)**						
Alpha/Bravo	30 (97)	30 (97)	17 (100)	15 (100)	0.99	0.66
Charlie/Delta	1 (3)	1 (3)	0 (0)	0 (0)		

Retreatment was performed in five teeth at 6 months, in two teeth at 12 months and in three teeth at each of the two next follow-ups (Table 2). Up to 12 months, most retreatments were for sealants and preventive resin restorations, with only two being for composite resin restorations. Regarding changes in initial treatment, only one sealant had to be replaced with a composite resin and one composite restoration replaced with a SSC at the 12-month follow-up. Minor alterations observed with time were not considered statistically significant.

**Table 2:** Evaluation of further treatment needs at tooth level during follow-ups

	Follow-up (months: mean; SD) 17.5 (6.25)					
	6 (*N* = 126)	12 (*N* = 126)	18 (*N* = 66)	24 (*N* = 59)	*p* value	Trend *p* value
16 (*n*, %)						
No change in treatment	29 (94)	29 (94)	14 (93)	13 (100)	0.95	0.63
Repeat of same treatment	2 (6)	0 (0)	1 (7)	0 (0)		
Sealant to composite resin	0 (0)	1 (3)	0 (0)	0 (0)		
Composite resin to SSC	0 (0)	1 (3)	0 (0)	0 (0)		
SSC to extraction	0 (0)	0 (0)	0 (0)	0 (0)		
26 (*n*, %)						
No change in treatment	33 (100)	33 (100)	16 (94)	14 (93)	0.99	0.88
Repeat or same treatment	0 (0)	0 (0)	1 (6)	1 (7)		
Sealant to composite resin	0 (0)	0 (0)	0 (0)	0 (0)		
Composite resin to SSC	0 (0)	0 (0)	0 (0)	0 (0)		
SSC to extraction	0 (0)	0 (0)	0 (0)	0 (0)		
36 (*n*, %)						
No change in treatment	29 (94)	30 (97)	16 (94)	16 (94)	0.98	0.51
Repeat or same treatment	2 (6)	1 (3)	1 (6)	1 (6)		
Sealant to composite resin	0 (0)	0 (0)	0 (0)	0 (0)		
Composite resin to SSC	0 (0)	0 (0)	0 (0)	0 (0)		
SSC to extraction	0 (0)	0 (0)	0 (0)	0 (0)		
46 (*n*, %)						
No change in treatment	30 (97)	29 (97)	16 (94)	14 (93)	0.99	0.66
Repeat or same treatment	1 (3)	1 (3)	1 (6)	1 (7)		
Sealant to composite resin	0 (0)	0 (0)	0 (0)	0 (0)		
Composite resin to SSC	0 (0)	0 (0)	0 (0)	0 (0)		
SSC to extraction	0 (0)	0 (0)	0 (0)	0 (0)		

### Correlations with patient and tooth-related factors

Table 3 presents the results of multinomial logistic models, with treatment as the outcome. After adjusting for the follow-up period, none of the tooth and patient-related factors seem to significantly affect the outcome (*p* values > 0.05). Although, it should be noted that gender, age at first presentation and involvement of incisors present a > 1 probability of causing failure. The corresponding probability for presence of caries and lesion severity is more than three in some cases. It should be mentioned though that the wide confidence intervals presented are mainly attributed to the small sample size.

**Table 3:** Multinomial logistic regression, reporting the effect of various patient and tooth-related factors on the outcome

Tooth treated	Covariate	OR and 95% CI	*p* value
#16	Gender (female vs male)	1.48 (0.23–9.42)	0.676
	Age at 1 st presentation (years)	0.84 (0.47–1.50)	0.559
	Severity	0.67 (0.16–2.87)	0.594
	Number of molars affected	0.59 (0.11–3.07)	0.527
	Incisors also affected	1.02 (0.47–2.19)	0.969
	Type of lesion		
	*Opacities*	0.88 (0.07–10.65)	0.922
	*Enamel breakdown*	0.73 (0.30–1.75)	0.482
	*Caries*	4.60 (0.71–29.88)	0.110
	Pulp involvement	1.01 (0.98–1.04)	0.553
#36	Gender (female vs male)	0.63 (0.10–4.00)	0.625
	Age at 1 st presentation (years)	1.17 (0.74–1.84)	0.504
	Severity	3.69 (0.42–32.47)	0.239
	Number of molars affected	0.42 (0.07–2.61)	0.350
	Incisors also affected	1.25 (0.83–1.90)	0.289
	Type of lesion		
	*Opacities*	1.63 (0.51–5.20)	0.406
	*Enamel breakdown*	1.00 (0.45–2.20)	0.999
	*Caries*	3.19 (0.50–20.45)	0.220
	Pulp involvement	0.99 (0.84–1.18)	0.943
#46	Gender (female vs male)	0.63 (0.24–1.65)	0.347
	Age at 1 st presentation (years)	1.17 (0.72–1.93)	0.525
	Severity	0.66 (0.08–3.24)	0.994
	Number of molars affected	0.31 (0.05–2.03)	0.222
	Incisors also affected	1.00 (0.63–1.57)	0.995
	Type of lesion		
	*Opacities*	1.13 (0.36–3.62)	0.832
	*Enamel breakdown*	0.43 (0.27–2.20)	0.993
	*Caries*	0.94 (0.33–2.70)	0.907
	Pulp involvement	1.02 (0.98–1.05)	0.404

## Discussion

MIH is an oral clinical entity of systemic origin and geographical variability, with severe consequences for the patient (Lopes et al., 2021). This along with the increased treatment and re-treatment needs, worsens patients’ oral health-related quality of life and in many cases increases the economic burden (Almuallem Z and Busuttil-Naudi, 2018). The present retrospective study attempted to evaluate long-term success of treatment modalities and the need for re-treatment on MIH-affected teeth of different severities and identify specific patient and tooth-related characteristics that are directly correlated to the treatment outcomes, increasing the strength of the results. Results showed an overall high success rate that underlines the quality of care and the importance of following standardised protocols. Direct and indirect restorations were equally successful, with none of the patient and tooth-related characteristics affecting the outcome. The need for re-treatment was low and mainly for minimally invasive adhesive restorations, such as fissure sealants and preventive resin restorations.

The above results should be explained within the context of the study’s limitations and the conclusions drawn interpreted with caution, highlighting the importance of the recorded information for future studies. Data were retrieved from a population seeking dental care for this condition in a university clinic, one of the two university clinics specialised for paediatric dentistry in the country. This means that a considerable number of children with MIH-affected teeth and high, as well as demanding treatment needs, are referred to and treated in these facilities. Although, given the retrospective nature of the study, one should consider the gaps in the recording of relevant information, such as whether the affected enamel was totally or partially removed, and what the specific restorative materials used was. These gaps are partially bridged as the clinicians involved follow a specific protocol in the clinic, guided by lesion characteristics and based on the available evidence.

Regarding the clinical features of MIH-affected teeth in the given sample, a majority of the patients presented with severely affected molars presenting enamel breakdown and active caries. This is in accordance with previous studies that have shown that children with MIH had higher DMFT scores, which can be attributed to the developmental defects of the dental structures and the inadequate oral hygiene due to the hypersensitivity that act as a deterrent (Mazur et al. 2023). Rapid caries development can be seen in severely affected enamel due to bacterial invasion in areas where there is structural break down due to masticatory forces (Fagrell et al., 2010; Heyeraas and Bergreen, 1999). These parameters plus the fact that children in need for complex treatments are seeking dental care late, as most of the participants of the present study did, contribute to the high number of children with caries in severe MIH-affected teeth.

High was the percentage of patients reporting hypersensitivity, a fact that can be attributed to the high prevalence of severe characteristics and the increased caries incidence in the sample. Despite the lack of a specific explanation, the role of the increase in local tissue pressure and inflammatory mediators (Özgür et al. 2022), the insufficient isolation of the pulp, as hypomineralised tissues are poor insulators (Discepolo et al., 2011), and the bacterial invasion due to decreased mineral density and increased porosity (Rodd et al. 2021) is highlighted. These effects cause chronic stress to the pulp, promote inflammation, increase innervation and hypervascularity of the pulp and change the pH at the peripheral tissues. These reactions are translated into spontaneous pain, a decreased pain threshold and an increased responsiveness to noxious stimuli clinically directly affecting patient’s behaviour during treatment. Clinical implication of the above is teeth that are difficult to be adequately anaesthetized and patients that become extremely stressed and uncooperative. Presence of pre-operative and intra-operative hypersensitivity in these patients has been previously highlighted, with studies underlining its presence in severely affected teeth and in disintegrated molars immediately after eruption (Raposo et al. 2019; Linner et al. 2021; Özgül et al. 2022).

To overcome the effects of intra-operative hypersensitivity, recent research has supported the use of inhalation sedation (Fayle 2003; William et al. 2006) and different local anaesthesia adjuncts, such as intraligamentous and intraosseous (Bigby et al. 2006; Jadhav and Mittal 2016) anaesthesia to reduce pain threshold. Pre-operative techniques including desensitising agents and anti-inflammatory drug administration before the appointment have also been suggested (Discepolo et al. 2011; Vicioni-Marques et al., 2022). This falls within the protocols followed in the department of paediatric dentistry (NKUA), accompanied by pre-treatment administration of anti-inflammatory drugs. The logic behind this is that anti-inflammatory drugs suppress the conduction of mediators, decreasing the inflammation and allowing anaesthesia to be administered in the tissues adequately and effectively, decreasing overall pain sensation. This might also explain the higher success rate in relation to more adequate anaesthesia; the more comfortable the patient is and the better the clinical outcome.

In the present study, more invasive treatment protocols were chosen as most affected molars presented enamel breakdown and caries. In the majority of the cases, to avoid further hard tissue loss, the placement of SSC was chosen showing an overall high success rate. This is in accordance with previous studies, reporting rates that range between 86 and 100% for severely affected permanent molars with two or more affected surfaces and cuspal involvement that had been treated with indirect restorations (Weber et al., 2021). De Farias et al. (2022) reported that SSC showed greater therapeutic effectiveness than composite resins, over a period of 24 months, even supporting no tissue removal through a micro-invasive approach. These high rates in combination with the decrease in reported hypersensitivity underline the outperformance of the material that can be considered as a low-cost alternative when compared to other indirect restorations (Kotsanos et al. 2005; Elhennawy and Schwendicke 2016; Rolim et al. 2021; de Farias et al. 2022).

Within the context of the high success rates of indirect restorations, a small number of studies reported promising survival rates for composite and ceramic onlays on MIH-affected teeth, showing sufficient marginal adaptation (Dhareula et al. 2018; 2019; Linner et al. 2020). This high success rate might be contributed to the fact that all preparations were made with margins in sound enamel. Although, in the systematic review by Weber et al. (2021), it was mentioned that this technique is mostly indicated for severe MIH-affected teeth as it, unlike SSC, requires extensive tissue removal to make space for the required thickness of the restorative material. It was also mentioned that in these types of restorations, eruption status, patient’s age, compliance and cost should be also considered during treatment planning. In our study, despite the indication, the main reason for not choosing this type of treatment was financial issues that patients visiting the above clinic face.

Composite resin restorations also demonstrated a high long-term success rate in the present study. This is in accordance with two recent systematic reviews (Weber et al. 2021; Somani et al. 2022) concluding that resin composites after complete or partial removal of affected enamel can be used for restoring MIH-affected teeth regardless of the lesion severity and the adhesion system used. With a long-term stability with around 5 years of median survival rate in MIH-affected teeth and the high success rate that ranges between 74 and100% up to 4 years post-treatment, they are considered ideal direct restorative material (Elhennawy and Schwendicke 2016; Rolim et al. 2021; Lygidakis et al. 2022). The high success rates recorded can be attributed to the fact that composite resin is the material of choice in less severe cases with no cusp involvement and mainly only one affected surface. Although, earlier de Souza et al. (2017) and Kotsanos et al. (2005) recorded lower rates that ranged from 59 at 18 months to 75% at 52 months. These differences can be attributed to differences in the lesion characteristics and the restorative techniques used, since, in the study by de Souza et al. (2017), the teeth were initially restored with a glass ionomer cement, which was then replaced with the final composite restoration.

Other materials that have been used in the literature as direct restorations for the treatment of MIH-affected teeth are glass ionomer cements (GICs). Evidence shows a high overall survival rate of the material that exceeds 85% for hypomineralised young permanent teeth 24 months post-treatment (Durmus et al. 2021). Their success is attributed to their ability to bind to calcium in enamel structure, whilst being easily applicable make it an ideal option for the treatment of partially erupted molars showing post-eruptive break down and/or patients with difficulties in behavioural management (Fragelli et al. 2015; Casagrande et al. 2017) In the present study, none of the affected teeth was restored with this material due to the extent of the tooth structure loss.

Micro-invasive techniques in the present study could not be easily adopted and sealants were, therefore, chosen in very few cases. In the literature, there are also few studies reporting a 22% annual failure rate after a follow-up time of 18 months, rate slightly lower than that calculated for healthy teeth (Fragelli et al. 2015). A recent systematic review underlined that they are predominantly used for fully erupted teeth that present mild lesions (Somani et al., 2022), to decrease potential for caries lesion initiation and enamel breakdown (Lygidakis et al., 2009; Fragelli et al. 2015).

Finally, in the present study, extractions were performed in only 8% of the MIH-affected teeth and involved unrestorable teeth, with extensive hard tissue loss and in most cases symptomatic with active caries and pulp involvement. This type of treatment has been previously justified by the long-term clinical and economic consequences they pose in terms of orthodontic treatment needs and more frequent follow-ups (Elhennawy et al. 2017). In the literature, there are very few studies reporting on extraction of MIH-affected teeth with space closure being the main outcome measured. Results showed great variation on success rates, with complete space closure not being achieved in all cases. This variation was attributed to the small sample, the wide age range at extraction and the specific orthodontic needs, supporting the need for orthodontic consultation before any extractions is planned.

In the present study, re-treatment needs were relatively low and mainly in cases of direct resin restorations. This is in accordance with previous studies reporting that despite MIH-affected children being in need and receiving more dental treatment, restored molars remain within short re-treatment cycles (Elhennawy et al. 2017). This can be attributed to the fact that changes in the ultrastructure are evident in healthy areas of affected molars and therefore, adhesion is often compromised even after complete removal of the hypomineralised tissue (Bozal et al. 2015). Sönmez and Saat (2017) reported that failures occurred predominantly in restorations where hypomineralised and/or decayed tissue has been selectively removed, with the highest success rates occurring in teeth where complete removal of all affected enamel has been performed. Lagarde et al. (2020) have also shown in vitro poorer adhesion and reduced bond strength in MIH-affected teeth treated with a less invasive approach.

Finally, results from the present study reported no significant change in treatment outcomes when demographic characteristics and clinical features were considered as confounding factors (*p* values > 0.05). Previous studies also agreed that treatment outcome was not affected by variables such as gender, dental arch and severity of MIH-affected lesions (Durmus et al. 2021). Although there are studies reporting that the success of treatment protocols depends on severity of the lesions, patient’s caries risk, cooperation and age at presentation (de Souza et al. 2017). These differences might be attributed to the different characteristics of the sample in each study, such as age, caries risk and specific characteristics of the MIH-affected lesions and underline the need for more well-designed studies.

Therefore, despite the very high long-term success rates for both direct and indirect restorations up to 24 months post-treatment shown in the present study and in previous reports (Somani et al. 2022; Gatón-Hernandéz et al. 2020), slight differences exist and are attributed to the various criteria used for the evaluation of the restoration which consider different subjective clinical and/or radiographic criteria to determine success or failure. Although, it was agreed that effective management of MIH-affected teeth in paediatric patients demands prudent and wise treatment planning. An individualised approach is essential to meet the needs and expectations of each patient, optimising outcomes given the wide spectrum of lesion severity, the diversity of clinical manifestations and overall oral health status. This underlines the need for further studies with bigger sample sizes and longer follow-ups and neutralisation of confounding factors to develop specific treatment protocols.

## Conclusions

- Management of MIH-affected teeth with direct and indirect restorations was successful, presenting low re-treatment needs.
- Severely affected teeth, presenting with combination of lesions and extensive carious lesions, are mainly treated with SSC and composite resins, modalities that showed high success rates even 24 months post-treatment.
- Need for re-treatment was mainly indicated for minimally invasive adhesive restorations, such as fissure sealants and preventive resin restorations.
- None of the patient and tooth-related characteristics seem to affect the outcome.
- The extent of carious destruction indicates the importance of prevention and early diagnosis in intervention of MIH-affected teeth.

## Data Availability

The datasets used and/or analysed during the current study are available from the corresponding author on reasonable request.
