# Correction: Long-term evaluation of the success rate of different treatment modalities on MIH-affected teeth

**DOI:** 10.1007/s40368-025-01138-y

**Published:** 2025-11-15

**Authors:** K. Seremidi, K. Petroleka, N. Kraemer, S. Gizani

**Affiliations:** 1 Department of Paediatric Dentistry, School of Dentistry, National and Kapodistrian University of Athens, Athens, Greece; 2 Department of Paediatric Dentistry, Justus Liebig University, Giessen, Germany

**Correction: European Archives of Paediatric Dentistry** 10.1007/s40368-025-01106-6

The article [Long-term evaluation of the success rate of different treatment modalities on MIH-affected teeth], written by [K. Seremidi 1 · K. Petroleka 1 · N. Kraemer2 · S. Gizani1], was originally published Online First without Open access. After publication, the author decided to opt for Open Choice and to make the article an Open Access publication. Therefore, the copyright of the article has been changed to ©[The Author(s)] [2025] and this article is licensed under a Creative Commons Attribution 4.0 International License, which permits use, sharing, adaptation, distribution and reproduction in any medium or format, as long as you give appropriate credit to the original author(s) and the source, provide a link to the Creative Commons licence, and indicate if changes were made. The images or other third party material in this article are included in the article’s Creative Commons licence, unless indicated otherwise in a credit line to the material. If material is not included in the article’s Creative Commons licence and your intended use is not permitted by statutory regulation or exceeds the permitted use, you will need to obtain permission directly from the copyright holder. To view a copy of this licence, visit http://creativecommons.org/licenses/by/4.0/.

The original article has been corrected.

**Funding Open Access** Open access funding provided by HEAL-Link Greece.

